# An improved SIFT algorithm for registration between SAR and optical images

**DOI:** 10.1038/s41598-023-33532-1

**Published:** 2023-04-18

**Authors:** Wannan Zhang, Yuqian Zhao

**Affiliations:** School of Automation, Central South University, Changsha, China

**Keywords:** Engineering, Mechanical engineering

## Abstract

Aiming at improving the performance of scale invariant feature transform (SIFT) algorithm during the registration of optical and synthetic aperture radar (SAR) images, a new SIFT algorithm is proposed. Firstly, the nonlinear diffusion scale space of optical and SAR images is constructed by using nonlinear diffusion filtering, the uniform gradient information is calculated by using multi-scale Sobel operator and multi-scale exponential weighted mean ratio operator respectively. Then, after removing the first layer of the scale space with the image blocking strategy, the scale space is partitioned, and Harris feature points are extracted on the basis of consistent gradient information to obtain stable and uniform point features. Descriptors are constructed based on gradient position and direction histogram templates and normalized to overcome nonlinear radiation differences between images. Finally, the correct matching point pairs are obtained by using bilateral fast approximate nearest neighbor (FLANN) search matching method and random sampling consistent (RANSAC) method, and the affine transformation model parameters are obtained. Compared with the other two algorithms, the CMR of this algorithm is improved by 80.53%, 75.61% and 81.74% respectively in the three groups of images, and the RMSE is reduced by 0.6491, 1.0287 and 0.6306 respectively.

## Introduction

With the development of sensor technology, the types of remote sensing images are increasing rapidly at the same time. There are obvious differences in the spectrum, spatial resolution, imaging methods, etc. of a single sensor, and there are also differences in the ground information obtained. It is difficult to meet the needs of information diversification by using only one image, and the surface object information obtained from multi-source remote sensing images has a certain degree of complementarity. Therefore, studying the analysis and utilization of different types of remote sensing images can provide more comprehensive information for surface monitoring^1^. The spatial resolution of optical images is high, and the information contained therein is easy to interpret, but the imaging results are vulnerable to weather and light conditions; SAR images have all-weather and all-weather imaging characteristics, can penetrate cloud, fog and other media, and have strong complementarity with optical images. It has broad application prospects in military and civilian fields^2^, but the presence of speckle noise will affect the information resolution of SAR images. Therefore, it is of great significance to study the registration of optical and SAR images in image processing and analysis, image fusion, computer vision and other fields^3^.

Registration algorithms can be divided into gray based registration algorithm and feature-based registration algorithm. The principle of gray-scale based registration algorithm is simple, but it is less robust to images with large gray-scale differences such as optical and SAR images, and changes in scale and rotation have a large impact on the algorithm, which is not suitable for the registration of optical and SAR images^4^. Feature based registration algorithm has good adaptability to gray difference, rotation and scale change, such as SIFT^5^ and accelerated robust feature (SURF) calculation^6^. Among them, SIFT algorithm is widely used in optical image registration^7^. Yu et al.^8^ used the combination of spatial feature detection and local frequency domain description to construct the rotation invariant amplitude descriptor based on the direction histogram, and realized the registration of optical and SAR images. Sedaghat et al. proposed an optimization scheme to solve the problem of uneven distribution of feature points detected by SIFT algorithm in optical remote sensing image registration, which extracted uniformly distributed feature points by using the constraint information of feature points in scale and spatial position^9^. Subsequently, Dellinger et al. applied SIFT algorithm to SAR image registration and solved the impact of speckle noise inherent in SAR images on SIFT algorithm by introducing a new gradient definition^10^. Dellinger et al.^11^ proposed a SAR-SIFT algorithm for SAR image registration. By introducing the multi-scale exponential weighted mean ratio (ROEWA) operator to calculate the gradient of SAR image, the feature points were detected in Harris scale space, and good registration results were achieved. Based on the SIFT algorithm, Ma and other scholars proposed a gradient calculation method that could overcome the nonlinear gray difference between images, and used the spatial position, scale and direction information of feature points to match multi-modal images, which was successfully applied to the registration of visible and SAR images. The proposed algorithm was named PSO-SIFT^12^. Wang et al.^13^ used anisotropic diffusion filtering (SRAD) to construct the anisotropic scale space, which reduced the influence of noise on feature extraction and effectively improved the registration accuracy of SAR images. Dellinger et al. Xiang et al. used two different gradient operators for optical and SAR image respectively to extract the consistent gradient information of the two types of images, and adopted multi-scale Harris corner detector to solve the sensitivity of traditional Harris detector to scale change. The proposed algorithm is robust to large scale and rotation differences between optical and SAR images^14^. Fan et al. proposed an algorithm with high accuracy for the registration of optical and SAR images. The nonlinear diffusion operator was used to extract evenly distributed feature points, and the phase consistency operator was used to construct descriptors, so as to improve the accuracy of the registration algorithm. However, this algorithm is highly dependent on the image structure information. The registration effect of images containing less structural information is not ideal^15^. Divya et al.^16^ proposed a SIFT algorithm based on structure tensor, and used the descriptor constructed by the algorithm for SAR image registration, which can increase the number of correctly matched point pairs and improve the position accuracy of registration. Ye et al. proposed a novel feature detector that combined point features and patch features to effectively improve the repetition rate and matching accuracy of feature point pairs between visible and SAR images^17^. Reference^18^ overcame the problem of image gray scale and improved the registration accuracy by improving SIFT gradient definition. The method is based on neural network which usually adopts the end-to-end network to realize image registration. Literature^19^ realized the process of image registration by learning modal invariant features and improved the registration accuracy.

All the above algorithms improve the registration accuracy and speed of optical and SAR image in a fixed range degree, but there are still some limitations. On the one hand, the nonlinear difference between optical and SAR images is large, so it is difficult to extract highly repeatable features with the same feature detector. On the other hand, these algorithms are not robust to airborne SAR images with large noise. To solve these problems, we propose an improved SIFT algorithm for registration between SAR and optical images. Firstly, the nonlinear diffusion scale space of optical and SAR images is constructed by using nonlinear diffusion filtering, and the uniform gradient information is calculated by using multi-scale Sobel operator and multi-scale exponential weighted mean ratio operator respectively. Then, after removing the first layer of the scale space with the image blocking strategy, the scale space is partitioned, and Harris feature points are extracted on the basis of consistent gradient information to obtain stable and uniform point features. Descriptors are constructed based on gradient position and direction histogram templates and normalized to overcome nonlinear radiation differences between images., Finally, the correct matching point pairs are obtained by using bilateral fast approximate nearest neighbor (FLANN) search matching method and random sampling consistent (RANSAC) method, and the affine transformation model parameters are obtained.

## Proposed method

### Nonlinear diffusion scale space

SIFT algorithm uses Gaussian filter to construct scale space, but the edge retention of Gaussian filter is poor which results in fuzzy natural boundary of objects. To solve this problem, the scale space is constructed by nonlinear diffusion filtering^20^, and the nonlinear diffusion equation can be expressed as:

1 $$ L_{t} = div[p(m,n,t)\nabla L] = p(m,n,t)\nabla L + \Delta p\nabla L $$

where p(m, n, t) is the diffusion function, div is the divergence operator, and ∇ and δ are the gradient and Laplace operators, respectively. Equation (1) is an anisotropic diffusion equation. When p(m, n, t) is a constant, it can be simplified to an isotropic diffusion equation:

2 $$ L_{t} = {\text{p}}(m,n,t)\nabla L $$

In order to make p(m, n, t) vary with the local features of the image, the function p(m, n, t) is expressed as:

3 $$ p(m,n,t) = g[||\nabla L(m,n,t)||] $$

where, ‖ ‖⋅ is modular operation, ∇L(m, n, t) is the gradient after Gaussian filtering, and g (·) is the edge function. It can not only preserve the edge, but also sharpen the brightness edge when g (·) is between 0 and 1. With the increase of scale, the edge details of the Gaussian scale space are lost more. The nonlinear diffusion scale space can effectively retain the edge detail features, and the blur degree between the images is also low, so the feature extraction effect is better which is conducive to the subsequent consistency gradient calculation.

### Partitioning strategy

The uneven distribution of feature points is easy to lead to the final successful matching feature points too concentrated, which is not conducive to the subsequent geometric transformation model. In order to make the feature points evenly distributed on the whole image to obtain a more accurate transformation model and higher registration accuracy, the segmentation strategy was introduced in the scale space. The main purpose of feature extraction stage is to obtain a sufficient number of relevant point features uniformly distributed in the image and scale space, while the block strategy obtains uniform point features in the whole space by extracting the feature points of each regular grid. According to the idea of block strategy^21^, a block strategy for scale space is designed. First, the total number of extracted feature points is specified. Then, each layer image is divided into regular grid cells and the number of feature points of each grid cell is determined. Finally, the first layer of the scale space is eliminated, and the feature points extracted by blocks are corresponding to the corresponding positions of the original scale space.

The position of each block is determined according to the size and number of the block, so as to determine the position of the feature points in the original scale space. The first layer of image in scale space is the original resolution image, and most feature points extracted from this layer are speckled noise, while the randomness of speckled noise will affect the actual feature points nearby, resulting in the mismatching of images^22^. In order to solve this problem, the feature extraction operation of the first layer of scale space is eliminated, and the image of this layer is not divided into blocks, so as to speed up the image registration speed to a certain extent. The segmentation strategy determines the segmentation form of the image according to the size of the image, and determines the segmentation mesh size of each layer according to the relationship between the scale space coefficients, and can be adjusted according to the actual application requirements, which speeds up the algorithm to a certain extent.

### Master direction assignment and descriptor construction

After the feature points are detected, the location information of the feature points is first used to correct the location of the detected feature points, and then the GLOH descriptor is used to describe the feature. GLOH algorithm divides the circular neighborhood of feature points into three concentric circles, and divides the two peripheral concentric circles into 8 equal parts, and the central circle remains unchanged, which is divided into 17 sub-regions. The gradient direction of 0°–180° in each small region is divided into 8 directions, and the histogram of the gradient direction of the feature point is obtained statistically. Then, the peak value of the histogram represents the main direction of the feature point. This structure belongs to the isotropic average and can increase the robustness of the descriptor.

### Feature matching and mismatching elimination

FLANN algorithm is a binary feature matching method, which realizes feature matching by calculating the nearest neighbor points of feature points at different Euclidean distances. A FLANN search and matching method is designed, in which I_1_ is SAR image and I_2_ is optical image. Points q and m are the nearest neighbor feature points and the second nearest neighbor feature points of the midpoint p of I_1_ on I_2_, respectively, and the corresponding feature vectors are F_q_, F_m_ and F_p_. r and s are the nearest neighbor feature points and the second nearest neighbor feature points of the middle point n of I_2_ on I_1_, respectively. The corresponding eigenvectors are F_r_, F_s_ and F_n_. The main steps are as follows:

1. Formula (4) is used to calculate d_pq_ and d_pm_ of Euclidean distance between point q and m and point p, and d_nr_ and d_ns_ of Euclidean distance between point r and s and point n:
  4 $$ \left\{ {\begin{array}{*{20}c} {d_{pq} = \sqrt {\sum\limits_{i = 1}^{64} {[F_{p} (i) - F_{q} (i)]^{2} } } } \\ {d_{pm} = \sqrt {\sum\limits_{i = 1}^{64} {[F_{p} (i) - F_{m} (i)]^{2} } } } \\ \end{array} } \right.,\left\{ {\begin{array}{*{20}c} {d_{nr} = \sqrt {\sum\limits_{i = 1}^{64} {[F_{n} (i) - F_{r} (i)]^{2} } } } \\ {d_{ns} = \sqrt {\sum\limits_{i = 1}^{64} {[F_{n} (i) - F_{s} (i)]^{2} } } } \\ \end{array} } \right. $$
2. Calculate the distance ratio R_1_ and R_2_:
  5 $$ R_{1} = \frac{{d_{pq} }}{{d_{pm} }},R_{2} = \frac{{d_{nr} }}{{d_{ns} }} $$
3. Judge the relationship between distance ratio R_1_, R_2_ and threshold Th respectively: if R_1_ < T_h_, point p and q are matched successfully; otherwise, the matching fails. If R_2_ < T_h_, point n and r are matched successfully; otherwise, the matching fails.
4. The feature point pairs with the same matching results of the two searches are retained, and the one-to-one feature matching results of SAR and optical images are obtained.

RANSAC algorithm has high robustness and is often used to eliminate mismatched point pairs. Select 4 pairs of initial matching point pairs randomly from the pre-matching point pairs to calculate the affine transformation model parameters, then calculate the distance between the remaining matching point pairs and the pre-matching point pairs after coordinate transformation, and eliminate the wrong matching point pairs through several sampling calculations to achieve fine matching.

## Experimental results and analysis

**Figure 1:**
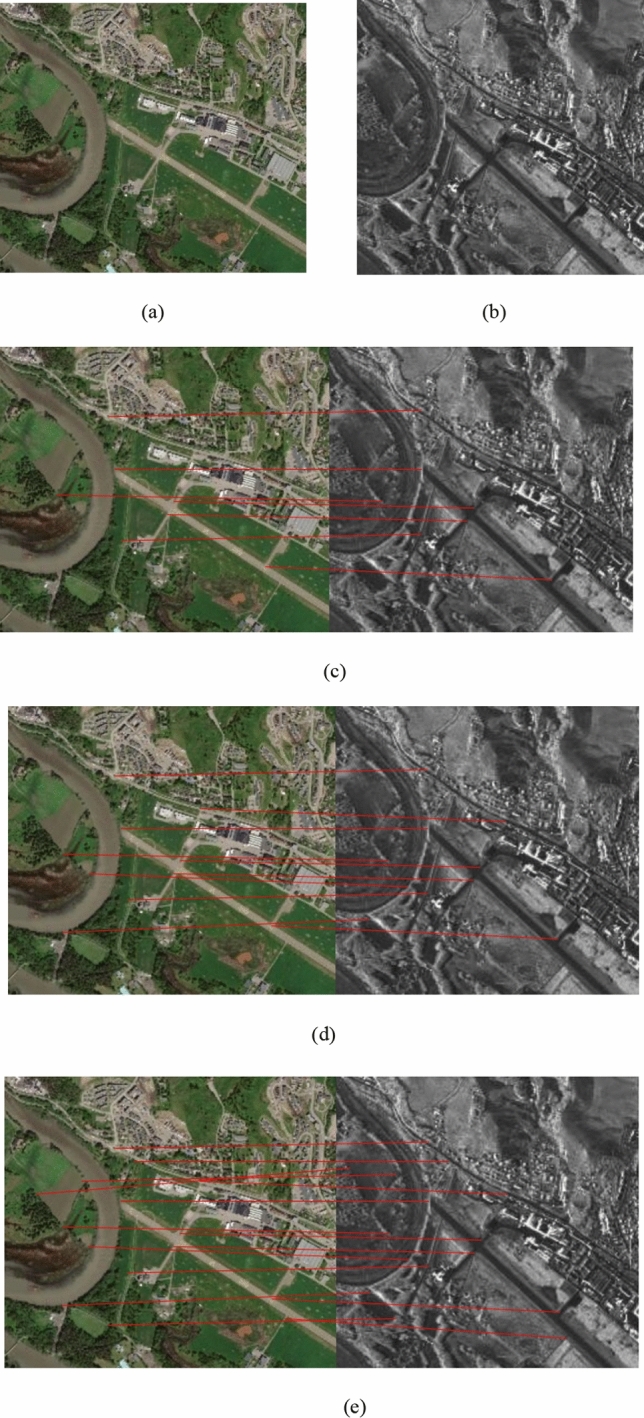
(**a**) Optical image; (**b**) SAR image; Matches found in pair 1 using (**c**) PSO-SIFT, (**d**) OS-SIFT, and (**e**) the proposed method. The reference image is shown on the left and the sensed image on the right.

**Figure 2:**
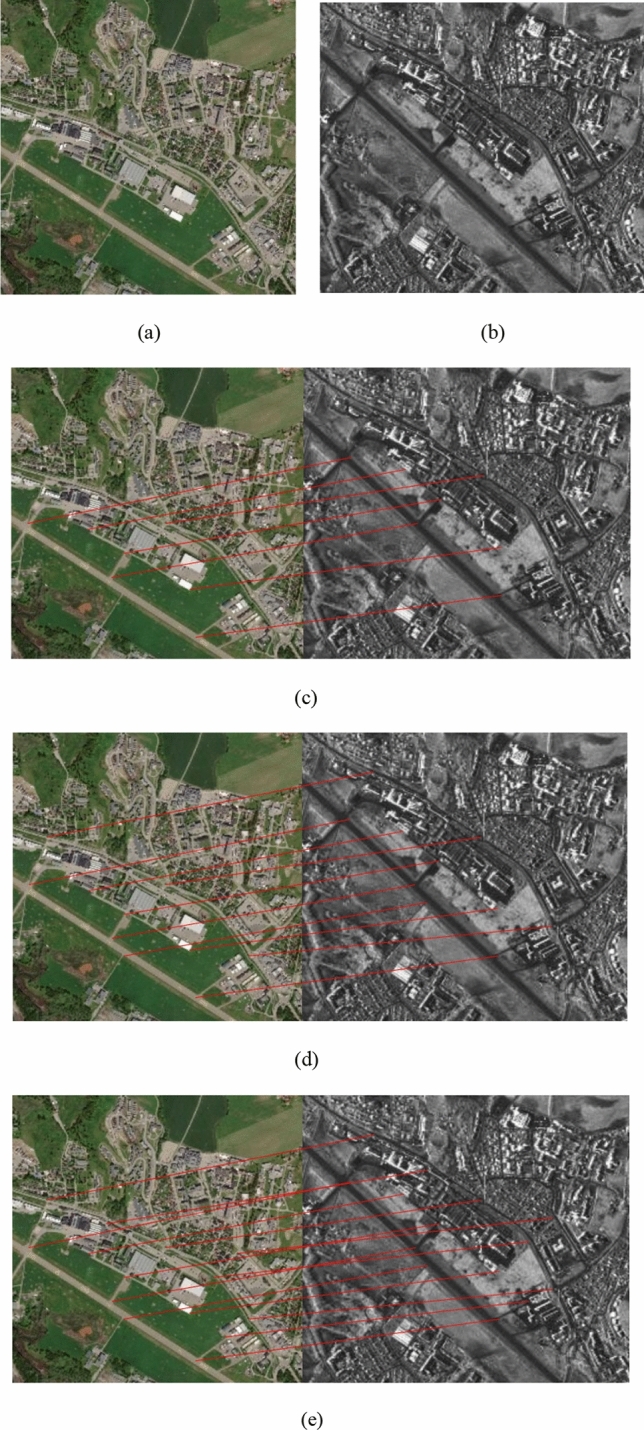
(**a**) Optical image; (**b**) SAR image; Matches found in pair 2 using (**c**) PSO-SIFT, (**d**) OS-SIFT, and (**e**) the proposed method. The reference image is shown on the left and the sensed image on the right.

**Figure 3:**
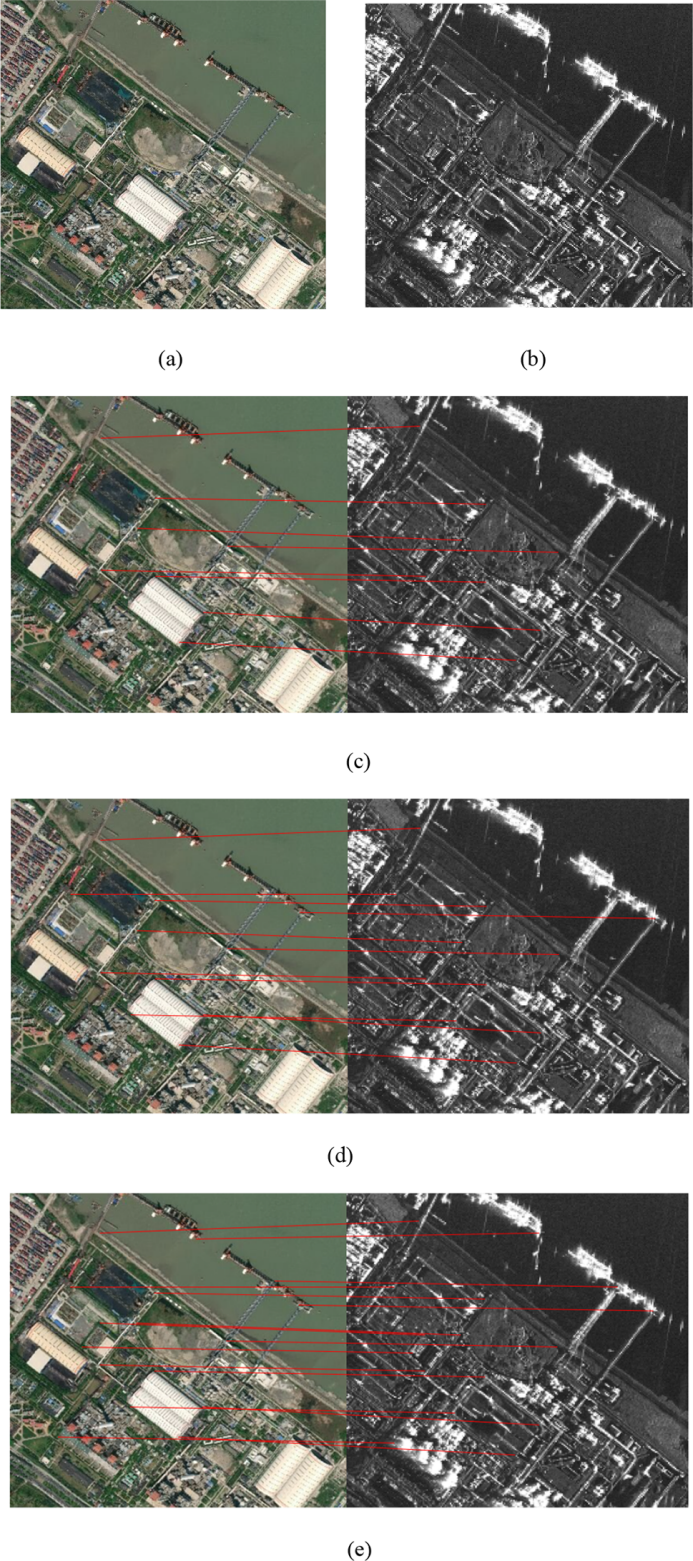
(**a**) Optical image; (**b**) SAR image; Matches found in pair 3 using (**c**) PSO-SIFT, (**d**) OS-SIFT, and (**e**) the proposed method. The reference image is shown on the left and the sensed image on the right.

To evaluate the performance of the proposed method, three pairs of SAR and optical images are experimented. The experiments are compiled with Python3.6, and the network is built through the deep learning framework of Pytorch1.3, and the corresponding CUDA10.0 and cudnn7.0 are configured for GPU acceleration. The test data consists of different characteristics including different resolutions, incidence angles, seasons etc. The dataset description is shown in Table 1. Experimental results are shown in Figs. 1, 2 and 3 and Table 2. Figures 1, 2 and 3 are generated by the software Python3.6, Python Release Python 3.6.10 | Python.org.

**Table 1:** Detailed description of dataset.

Image no	Image source	Size/(pixel × pixel)	Spatial resolution/m	Date	Location
1	TerraSAR-X	580 × 520	2.5	07/2018	Urban area
	Google Earth	580 × 520	3	05/2017	
2	TerraSAR-X	650 × 500	3	12/2010	River area
	Google Earth	650 × 500	3	09/2012	
3	TerraSAR-X	550 × 460	2	10/2018	Suburb area
	Google Earth	550 × 460	3	04/2018

**Table 2:** Quantitative comparison of the proposed method with other SIFT-based algorithms.

Image no	Method	CMR/%	RMSE/pixel
1	PSO-SIFT	72.56	1.1741
	OS-SIFT	78.02	1.0919
	Proposed	80.53	0.6491
2	PSO-SIFT	64.94	1.4496
	OS-SIFT	69.83	1.3581
	Proposed	75.61	1.0287
3	PSO-SIFT	70.63	0.8983
	OS-SIFT	75.95	0.8356
	Proposed	81.74	0.6306

To quantitatively evaluate the registration performances, we adopt the root-mean-square error (RMSE) between the corresponding matching keypoints, and it can be expressed as

6 $$ {\text{RMSE}} = \sqrt {\frac{1}{n}\sum\limits_{i = 1}^{n} {(x_{i} - x_{i} ^{\prime})^{2} + } (y_{i} - y_{i} ^{\prime})^{2} } $$

where (x_i_,y_i_) and (x_i_′,y_i_′) are the coordinates of the *i*th matching keypoint pair; n means the total number of matching points. In addition, correct matching ratio (CMR) is another effective measure which is defined as:

7 $$ CMR = \frac{correct\;Matches}{{correspondences}} $$

“correspondences” is the number of matches after using RANSAC, “correctMatches” is the number of correct matches after removing false ones. The results of quantitative evaluation for each method are listed in Table 2.

Analyzing the above experimental results, the following conclusions can be drawn: It can be seen from Table 2 that the correct matching rate obtained by the PSO-SIFT^23^ and OS-SIFT^24^ algorithms is relatively low. The proposed algorithm can obtain more and more uniform correct matching point pairs, which shows that this algorithm is better in suppressing the radiation difference between optical and SAR images. Compared with the other two algorithms, the CMR of this algorithm is improved by 80.53%, 75.61% and 81.74% respectively in the three groups of images, and the RMSE is reduced by 0.6491, 1.0287 and 0.6306 respectively. The reason is that the algorithm in this paper uses nonlinear diffusion filtering to build the scale space, which can effectively maintain the edge features of the image, and obtain an image with clear regional boundaries. In addition, this algorithm can also sharpen the image brightness edge, making the extracted features more effective, and laying the foundation for subsequent feature matching. After the block strategy is introduced, the distribution of feature points in the image is more uniform. Compared with the original algorithm, the uniform feature points can obtain a better transformation model, making the subsequent image registration better.

## Conclusion

In this paper, we propose an improved SIFT algorithm for optical and SAR image registration. Firstly, the nonlinear diffusion scale space of optical and SAR images is constructed by using nonlinear diffusion filtering, and the uniform gradient information is calculated by using multi-scale Sobel operator and multi-scale exponential weighted mean ratio operator respectively. Then, after removing the first layer of the scale space with the image blocking strategy, the scale space is partitioned, and Harris feature points are extracted on the basis of consistent gradient information to obtain stable and uniform point features. Descriptors are constructed based on gradient position and direction histogram templates and normalized to overcome nonlinear radiation differences between images., Finally, the correct matching point pairs are obtained by using bilateral FLANN search matching method and RANSAC method, and the affine transformation model parameters are obtained. Experimental results demonstrate its superior matching performance with respect to the state-of-the-art methods. However, the proposed algorithm has an impact on the running speed to some extent, because after the introduction of the block strategy, the algorithm needs to carry out block operation on the scale space when extracting the feature points, and after obtaining the feature points, the corresponding positions in the original scale space can be obtained. In the future, the algorithm can be further optimized to improve the operation speed.

## Data Availability

The datasets used and/or analysed during the current study available from the corresponding author on reasonable request.
